# Implementation and Feasibility of a Multidisciplinary Endocrine-Led Outpatient Clinic for Cancer Cachexia and Other Forms of Unintentional Weight Loss: A Real-World Observational Study

**DOI:** 10.3390/cancers18060946

**Published:** 2026-03-13

**Authors:** Anirudh Murthy, Morgan Simons, Anne Jablonski, Maurice Hurd, Alpana Shukla, Marcus D. Goncalves

**Affiliations:** 1 Department of Medicine, Weill Cornell Medicine, New York, NY 10065, USA; 2 Stony Brook Medicine, Stony Brook, NY 11794, USA; 3 Department of Medicine, New York University (NYU) Grossman School of Medicine, 435 E. 30th Street, New York, NY 10016, USA

**Keywords:** unintentional weight loss, cancer cachexia, muscle function, appetite stimulation, endocrinology

## Abstract

**Purpose:** Cachexia, characterized by involuntary weight loss, muscle wasting, and metabolic dysfunction, is prevalent in advanced cancer and chronic illnesses. Despite its impact, outpatient treatment models in the U.S. remain limited and unstandardized. Here, we aim to describe the structure, implementation, patient characteristics, and real-world clinical trajectories of a multidisciplinary clinic for cancer cache as well as other forms of unintentional weight loss clinic within an academic endocrinology practice. **Methods:** We conducted a retrospective observational cohort study of 103 patients referred to a single-center unintentional weight loss clinic over five years. Patients received comprehensive assessments (weight trajectory, nutrition status, 5× sit-to-stand test, handgrip strength) and personalized interventions including nutrition counseling, resistance training, and pharmacologic therapies. **Results:** Among 103 patients (median age 69.7 years; 53% male), 64% had cancer, while 36% were referred for non-malignant causes of weight loss or cachexia. Reduced appetite or food intake was reported in 43%, and functional impairment was common, with low handgrip strength in 47% and impaired 5× sit-to-stand performance in 79% of assessed patients. Systemic abnormalities were frequent, including elevated hs-CRP (57%), elevated neutrophil-to-lymphocyte ratio (43%), and hypoalbuminemia (26%). Among patients with available paired follow-up data, the median rate of weight change shifted from −0.5 kg/month prior to enrollment to 0.0 kg/month three months after the initial visit (*p* < 0.0001). Five-times sit-to-stand performance improved modestly at three months (*p* = 0.042), while handgrip strength was unchanged. Half of patients that engaged with the clinic returned for at least follow-up, but there was no identifiable difference between the population of patients that returned versus those that did not. **Conclusions:** A structured, multidisciplinary unintentional weight loss clinic in an endocrinology setting was associated with stabilization of weight and modest changes in physical function in this single-center cohort among patients who engaged in follow-up. These findings highlight the successful implementation of integrated outpatient care models and provide practice-based context for future interventions and therapeutic evaluations.

## Simple Summary

This study provides a detailed clinical, functional, and biochemical-based characterization of patients referred to a multidisciplinary, endocrinology-led unintentional weight loss clinic. Additionally, we describe observed weight trajectories and selected functional outcomes associated with care delivered in this setting. The results support the successful delivery of integrated outpatient care and characterize real-world clinical trajectories among patients who returned for follow-up.

## 1. Introduction

Cachexia is a multifactorial syndrome characterized by involuntary weight loss, skeletal muscle wasting, and metabolic dysregulation [1]. It commonly arises in the setting of chronic conditions such as heart failure, kidney disease, chronic obstructive pulmonary disease (COPD), and cancer, where the most data exist. The prevalence of cachexia in patients with advanced cancer is as high as 80% [2]. Cachexia holds clinical significance and demands serious consideration beyond the changes in body weight and composition, as cachexia is associated with impairment in functional status, reduced tolerance to therapy, and increased morbidity and mortality [3,4,5]. For example, patients with cachexia have increased complications from chemotherapy and surgery, poor therapeutic responses to immunotherapy, and worsened overall mortality, underscoring the need for focused attention on cachexia in both research and clinical practice [6,7].

Cachexia is clinically defined as unintentional body weight loss of 5% or more in 6 months or 2% or more, if the body mass index (BMI) is less than 20 kg/m^2^ [8]. Unintentional weight loss can result from reduced food intake, nutrient malabsorption, or increased energy expenditure. Reduced food intake can be found in 40–60% of patients with advanced cancer [9]. Malabsorption affects 30% to 80% of those with gastrointestinal (GI) malignancies [10], especially following radiation or surgery [11,12,13,14]. Less is known about high energy expenditure. Although elevations in energy expenditure have been reported in animal models, data supporting this mechanism of cachexia in humans remain limited [15]. These divergent mechanisms underscore the multifactorial nature of cachexia and the need for individualized diagnostic evaluation to inform targeted interventions.

Despite its high prevalence and important prognostic implications, the treatment of cachexia is limited by a lack of effective treatment. To date, there are no approved treatments specific to cachexia, outside of Japan, where anamorelin, a ghrelin receptor agonist, is available. In other countries, the current treatment approaches are supportive in nature and include nutritional therapy (calorie and protein goals), exercise (resistance training regimens), non-steroidal anti-inflammatory drugs (NSAIDs), steroid hormone receptor agonists (megestrol acetate and synthetic glucocorticoids), and other appetite stimulants (e.g., mirtazapine, olanzapine, cannabinoids, selective serotonin reuptake inhibitors (SSRIs) like paroxetine) [16]. While these agents can produce modest weight preservation, there is typically no improvement in functional status or survival [16]. Overall, there remains no consensus approach to care for patients with cachexia in day-to-day clinical practice.

Several institutions have developed dedicated cachexia clinical care programs to address this unmet need. Although cancer-associated cachexia is the most well-characterized and prevalent form of the syndrome, cachexia also occurs in a range of chronic diseases, and in routine clinical practice, referral patterns frequently include patients with cachexia as well as other forms of unintentional weight loss. These programs integrate pharmacologic symptom management, nutritional counseling, and physical rehabilitation, and have demonstrated improvements in symptom burden, appetite, emotional well-being, and quality of life despite limited changes in weight or lean mass [17,18,19,20,21]. Importantly, outcomes from these clinics highlight the value and deliverability of coordinated, individualized care in stabilizing functional decline and supporting patients and caregivers in the face of progressive disease.

While research has largely focused on the biological underpinnings of cachexia, less attention has been given to clinical coding, referral pathways, and scalable, multidisciplinary care models suitable for routine practice. Historically, clinical efforts have emerged primarily from oncology and palliative care, where cachexia is most visible due to its strong association with advanced cancer and its profound impact on outcomes. However, emerging mechanistic insights, particularly the role of the hormone GDF15, suggest that cachexia is, at least in part, an endocrine disorder. This opens the door for endocrinology to play a more central role in care delivery, given the specialty’s relevance to cachexia’s underlying biology.

Here, we present the structure, patient population, and outcomes of a multidisciplinary unintentional weight loss clinic embedded within an academic endocrinology division, offering a potential model for delivering care across diverse practice settings. This clinic was established to operationalize multidisciplinary care within routine clinical practice and to generate non-trial setting observational data, rather than to test a specific therapeutic hypothesis.

## 2. Methods

Data were collected through a manual review of the electronic medical record (EMR) for a 5-year period between 1 July 2018, and 31 July 2023, focused on all patients who were seen by Endocrinology for care of unintentional weight loss, cachexia, protein-calorie malnutrition, poor appetite, anorexia, sarcopenia, or muscle weakness as part of routine standard of care. Importantly, referral to the clinic was not restricted to patients with malignancy; individuals with non-malignant etiologies of unintentional weight loss or cachexia were also included. The study was reviewed by the Weill Cornell Medicine IRB and determined to qualify for exemption per the Code of Federal Regulations on the Protection of Human Subjects (45 CFR 46), under category 4 (ii). All procedures were conducted in accordance with the ethical standards of the institutional research committee and with the principles outlined in the Declaration of Helsinki. Because this was a retrospective chart review using existing de-identified data, the Weill Cornell Medicine IRB waived the requirement for informed consent.

### 2.1. Clinic Design and Approach

All clinical care was provided in the Endocrinology outpatient practice at Weill Cornell Medicine, located in New York City, NY, USA. Patients were evaluated and treated by two providers, an American Board of Internal Medicine (ABIM)-certified endocrinologist (MDG) and a nurse practitioner (AFJ) with expertise in endocrinology, obesity medicine, and reproductive health. Both were licensed to practice in New York State. Local providers were encouraged to refer patients for evaluation of unintentional weight loss if they experienced a weight loss of more than 5% over 6 months due to any suspected cause. At the time of presentation, a detailed body weight history was obtained, including self-reported recall of young adult weight, peak body weight and age, and weight values from 12, 6 and 3 months ago. All available body weight measurements were extracted from the EMR and discussed with the patient to identify discrepancies and errors. A GI review of systems was performed with specific inquiries into the presence or absence of anorexia, odynophagia, dysphagia, dysguesia, early satiety, abdominal pain, nausea, vomiting, diarrhea, constipation, and steatorrhea. The patient was asked about the number and size of meals, including a description of the types and amount of food consumed at each meal. The clinical team did not include a registered dietician nor a physical therapist.

As part of the clinical evaluation, all patients underwent a focused physical examination to assess skeletal muscle mass, strength, and the size of subcutaneous adipose tissue reserves. Visual inspection and palpation were used to evaluate muscle wasting, as well as fat loss. Muscle strength was graded on the standard 5-point Medical Research Council (MRC) scale in the deltoids, biceps, triceps, hip flexors, knee flexors, and knee extensors. Handgrip strength (HSG) was measured using a Jamar dynamometer and averaged over three trials with the dominant hand. When feasible, lower extremity performance was assessed using either the five-times sit-to-stand (5xSST) or the 30 s sit-to-stand (30sSST) test, selected based on safety and functional capacity [22,23].

EMR-based laboratory values were reviewed to rule out reversible causes of weight loss, including hypercalcemia, hyperglycemia, hyponatremia, uremia, hypogonadism, adrenal insufficiency, and hyperthyroidism. Abnormal results were further evaluated and managed in accordance with standard clinical practice. Laboratory data were also evaluated for evidence of malnutrition, including iron, folate, and vitamin B12 deficiency using complete blood counts and red cell indices, as well as vitamin D deficiency. When recent laboratory values were unavailable in the EMR, the tests were requisitioned. Inflammation was assessed using C-reactive protein (CRP) and white blood cell differential, including neutrophil and lymphocyte counts.

Nutritional counseling was provided to ensure adequate caloric and protein intake, with targets of >30 kcal/kg/day and >1.0 g/kg/day of protein, adjusted based on individual weight, comorbidities, and intake history. A sample 3-day meal plan was provided to each patient, designed to meet these goals using familiar and culturally appropriate foods. Dietary recommendations emphasized energy-dense, protein-rich meals and snacks. When food intake alone was insufficient to meet protein targets, supplementation with a whey protein isolate (20–30 g) was recommended with or without creatine monohydrate (5 g per day). Patients were encouraged to use a whey protein formulation with creatine monohydrate included. A daily multivitamin was recommended to help address potential micronutrient deficiencies and support overall nutritional adequacy. For patients who continued to lose weight after two weeks on the initial diet plan, caloric intake was increased by 300 kcal per day, with adjustments made incrementally until weight stabilization or gain was achieved.

After one month of treatment, patients who were unable to comply with the dietary plan were offered pharmacologic appetite stimulation. Low-dose mirtazapine was preferred when sleep disturbance was present, olanzapine when nausea was present, and a SSRI in cases of concurrent depressed mood. Steroid hormone receptor agonists (megestrol acetate and glucocorticoids) were avoided, given the high rate of deleterious adverse events, particularly on the endocrine system [16]. In some patients, anabolic-androgenic steroids (testosterone, oxandrolone) were used.

Patients were encouraged to begin a home-based resistance training program three days per week, consisting of a brief warm-up followed by lunges, wall push-ups, squats, overhead presses, bicep curls, and bent-over rows. A set of links to online videos demonstrating each movement was provided. Patients were instructed to perform these exercises using available household items such as hand weights, resistance bands, or water bottles to provide resistance, and to complete 3–4 sets of 10 repetitions each.

### 2.2. Statistical Analysis

We summarized baseline demographics, diagnoses and interventions as well as initial lab results using descriptive statistics. Because the data were not normally distributed, we used medians and interquartile ranges to describe continuous data, and frequencies to describe categorical data.

Weights at the four different time points, both summarized and as individual trajectories, were plotted using line graphs. In addition, we evaluated changes in weight trajectories before and after clinic entry to describe clinical patterns among patients with available follow-up. We selected a subset of 47 patients with good follow-up and paired weight measurements for analysis of pre- and post-clinic entry weight change rates. The rate of weight change was calculated by dividing the difference in weight by the time interval between visits. Wilcoxon signed-rank test was used to compare the rates of weight change between the three time intervals (i.e., 12 months before intervention to 6 months before intervention (interval I), 6 months before intervention to initiation of intervention (interval II), and initiation of intervention to 3 months after intervention (interval III)). Bonferroni correction was applied to compute adjusted *p*-values. Further Friedman analysis was performed on the rates of weight change across the three time intervals. For functional parameters, Wilcoxon signed-rank test was used to determine the significance of the change between baseline and three months after intervention.

Functional parameters (5xSST and maximum HGS) were evaluated at time of clinic entry and at 3 months and compared using Wilcoxon signed-rank test.

Percent change in weight during interval III was plotted against baseline laboratory values. Simple linear regression was additionally performed for visualization, and coefficients of determination (R^2^) were calculated. Associations were assessed using Pearson rank correlation coefficients.

In order to investigate differences between patients who returned for at least one follow-up visit and those who did not, we compared baseline demographics, rates of weight change, functional parameters, and baseline laboratory values. Baseline demographic variables were summarized using frequencies and compared between groups using Fisher’s exact test. Rates of weight change and functional measures were summarized as medians with interquartile ranges. Baseline laboratory values were visualized using violin plots to illustrate group distributions. Between-group differences in continuous variables were assessed using the Wilcoxon rank-sum test with Bonferroni correction where appropriate.

To better characterize the impact of individual management modalities on weight change, a regression analysis was performed. All paired and longitudinal analyses were restricted to patients who returned for follow-up and were intended to be descriptive and hypothesis-generating. All analysis and graphing were performed using R version 4.3.3 and Microsoft Excel.

## 3. Results

### 3.1. Patient Demographics and Characteristics

A total of 103 patients were treated. Most identified as white (65%) and male (53.4%). The median age was 69.7 years (IQR: 56.2–75.7 years). A majority of participants (53.9%) had a normal body mass index (BMI) (18.5–24.9 kg/m^2^) at the time of initial visit, but a plurality (30.9%) were severely malnourished according to prognostic nutritional index (PNI) (<40). Most patients had diagnosed cancer (64.1%). Of the patients with cancer, the most common types were lung (17.1%), pancreatic (12.9%), esophageal (11.4%), head and neck (5.7%), or prostate (5.7%). Most patients with cancer had diagnosed metastases (61.0%). Comorbidities included diabetes (24%) and thyroid disease (30%) (Table 1).

Symptoms of reduced food intake were common, as was objective evidence of impaired muscle function. Poor appetite and/or reduced food intake were reported in 44 (42.7%) patients. Of the 36 participants assessed for baseline, hand grip strength, 17 (47%) demonstrated low grip strength [24]. Among the 29 participants with recorded 5x sit-to-stand (SST) times, 23 (79%) performed below standard thresholds [25,26,27].

To better understand systemic factors that may contribute to these symptoms and the broader cohort phenotype, we assessed baseline hormonal, inflammatory, and nutritional biomarkers in the entire cohort. The median baseline testosterone level among males was 457 ng/dL [IQR: 267.5–564 ng/dL], with 21% meeting criteria for hypogonadism (testosterone < 264 ng/dL) [28]. The median baseline TSH was 2.0 mIU/L [IQR: 1.3–3.0 mIU/L], and 4% of participants were found to have hyperthyroidism (TSH < 0.4 mIU/L) [29]. The median hs-CRP concentration was 5.2 mg/L [IQR: 1.3–14.4 mg/L], with 57% demonstrating elevated levels (>3 mg/L) [30,31]. The median IL-6 level was 4.5 pg/mL [IQR: 2–5 pg/mL], and 8% of individuals had elevated levels of IL-6 (>7 pg/mL) [32,33]. The median neutrophil–lymphocyte ratio (NLR) was 3.28 [IQR: 2.1–5.4], and 43% had an elevated NLR (>3.83) [34,35,36]. Median albumin was 3.8 g/dL [IQR: 3.4–4.2 g/dL], with hypoalbuminemia (<3.5 g/dL) observed in 26% of participants [37].

Based on the clinical evaluation, providers identified contributing etiologies of weight loss and assigned specific ICD diagnoses accordingly, which guided individualized intervention strategies (Table 2). The most frequently documented ICD diagnoses included abnormal weight loss (R63.4, 67.0%), cachexia (R64.0, 45.6%), and anorexia (R63.0, 34.0%). The most common interventions included recommendations to increase protein and calories (76.7%), use of protein powder with or without creatine monohydrate (66.0%), and structured exercise videos (60.2%). A minority of patients received appetite stimulation therapy (29.1%), including olanzapine or mirtazapine, anabolic androgenic steroids (18.4%) like testosterone or oxandrolone, and/or pancreatic enzyme replacement (12.6%) for malabsorption.

### 3.2. Weight Trajectory over Time

**Figure 1:**
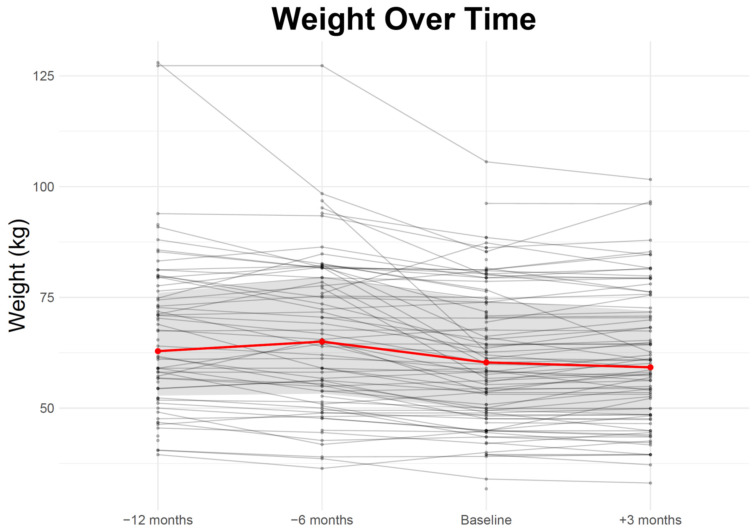
Weight Change Over Time. Scheme 12 months prior [N = 66], 6 months prior [N = 71], the intervention time point (reference) [N = 103], and 3 months post-intervention [N = 80]. Each thin gray line represents an individual participant’s trajectory. The thick red line shows the group median trend over time.

Median patient weight was 62.9 kg [IQR: 52.8–76.0 kg, N = 66] twelve months prior to clinical entry, 65.0 kg [IQR: 53.25–79.45 kg, N = 71] six months before entering the clinic, and 60.3 kg [IQR: 49.7–74.25 kg, N = 103] at the initial visit (baseline). Three months after baseline, the median weight was 59.2 kg [IQR: 48.5–71.9 kg, N = 80] (Table 3, Figure 1).

### 3.3. Rate of Weight Change over Time

**Figure 2:**
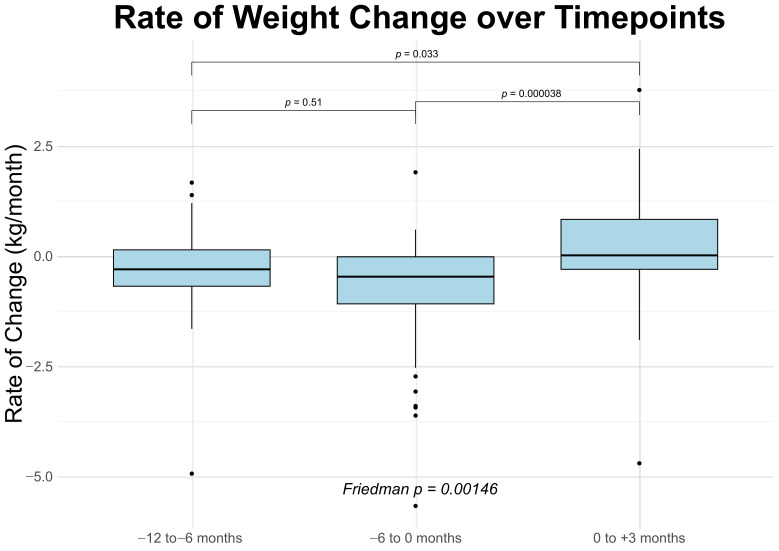
Rate of weight change over consecutive time intervals. Box plots show the distribution of individual rates of weight change (kg per month) across three intervals: −12 to −6 months, −6 to 0 months (intervention), and 0 to +3 months post-intervention (N = 47). Pairwise comparisons between intervals were assessed using Wilcoxon signed-rank test, with *p*-values indicated above brackets.

For patients with available paired measurements, the median loss of weight was 0.3 kg/month [IQR: −0.7–0.1 kg/month] over interval I; over interval II, patients lost a median of 0.5 kg/month [IQR: −1.1–0.0 kg/month]; and over interval III, patients’ median weight was unchanged (0.0 kg/month [IQR: −0.3–0.8 kg/month]) (Figure 2). Wilcoxon signed-rank analysis of the monthly rate of weight change between the intervals showed a statistically significant difference between interval II and interval III (*p* < 0.0001); likewise, there was a modest difference between interval I and interval III (*p* = 0.033). As expected, there was no significant difference between interval I and interval II (Table 4). Friedman analysis showed a *p*-value of 0.001 (Figure 2). Raw patient weights for this subset of patients are presented in Supplementary Table S1.

### 3.4. Functional Status over Time

**Figure 3:**
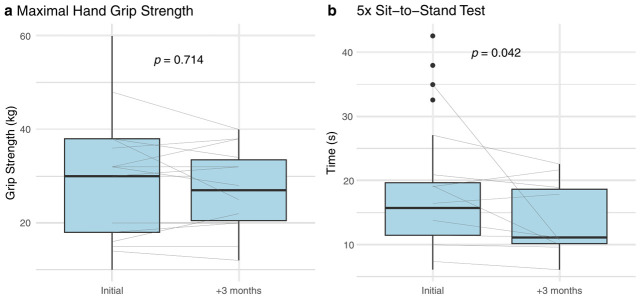
Change in Performance and Strength Over Time. (**a**) Performance on the 5x sit-to-stand test before and after 3 months. Box plots display the distribution of times (seconds) required to complete the 5x sit-to-stand test at the initial assessment and at 3 months (N = 36). Thin gray lines connect paired measurements for individual participants. The *p*-value reflects the statistical comparison of test performance between time points, showing a trend towards significance. Lower times indicate improved functional performance. (**b**) Hand grip strength at baseline and after 3 months. Box plots illustrate maximal hand grip strength (kg) measured at the initial assessment and at 3-month follow-up (N = 29). Each thin gray line represents the paired measurements for an individual participant. The *p*-value denotes the result of the Wilcoxon signed-rank testing between time points, showing no significance.

For patients with available paired measurements, there was a significant improvement in the 5xSST performance (*p* = 0.042) during interval III (Figure 3a) from 15.7 s [IQR: 11.5–19.7 s] at baseline to 11.1 [IQR: 10.2–18.7 s] after 3 months. There was no difference (30 kg [IQR: 18–38 kg] to 27 kg [IQR: 20.5–33.5 kg]) in strength as assessed by hand grip strength during interval III (*p* = 0.714) (Figure 3b).

### 3.5. Clinic Engagement and Follow-Up

Fifty-two patients (50%) did not return for a second visit. Among those who engaged in follow-up, 18 (17%) returned for one additional visit, 13 (13%) for two visits, and 10 (10%) for three or more visits. Documented reasons for non-return included disease progression, transition to hospice care, and competing medical priorities; however, reasons were unavailable for a substantial proportion of patients.

Baseline characteristics did not significantly differ between patients who returned for follow-up and those who did not, including race, sex, BMI, PNI, cancer and metastatic status, cancer type, or selected comorbid conditions (Supplementary Table S2). Similarly, there were no statistically significant differences in the rate of weight change between returners and non-returners across intervals I, II, and III.

Of note, during interval II (–6 months to baseline), there was a trend toward greater weight loss among patients who did not return for follow-up (–0.675 kg/month [IQR: –1.59 to –0.0958 kg/month]) compared with those who returned at least once (–0.417 kg/month [IQR: –0.993 to 0 kg/month]) (Supplementary Table S3)

Additionally, there was no significant difference between baseline lab values for patients that returned to clinic and patients that did not return to clinic (Supplementary Figure S1). Finally, there was no significant difference in 5xSST or hand grip strength between patients with and without follow up visits (Supplementary Table S4).

### 3.6. Association of Initial Biomarkers to Weight Change in Interval III

There was a weak linear relationship between the percentage of weight change over interval III and initial hs-CRP level (R^2^ = 0.17, *p* = 0.051); however, no association between weight change and serum IL-6 (R^2^ = 0.023, *p* = 0.48) (Supplementary Figure S2). No associations were found between weight change and other hematologic or endocrine parameters (Supplementary Figures S3 and S4).

### 3.7. Effect of Intervention on Weight Change

In exploratory regression analyses examining the relationship between individual management components and weight change during interval III, no intervention demonstrated a statistically significant independent association with weight trajectory. Participation in exercise videos showed a non-significant positive coefficient (β = +1.988, *p* = 0.079), while protein powder supplementation (with or without creatine) demonstrated a non-significant negative coefficient (β = −2.102, *p* = 0.113) (Supplementary Table S5). Given the observational design and the high likelihood of confounding by indication, these findings should be interpreted as descriptive and hypothesis-generating rather than reflective of treatment effect.

## 4. Discussion

Here we report data from a single-center, endocrinology-led unintentional weight loss clinic embedded within an academic outpatient practice and characterize the clinical, functional, and biochemical features of patients referred for care. This cohort demonstrated substantial heterogeneity in underlying diagnoses, mechanisms of weight loss, and systemic abnormalities, including impaired physical performance, reduced muscle strength, and markers of inflammation and hormonal dysregulation. These findings underscore the complexity of cachexia as encountered in routine clinical practice and highlight the need for integrated care models capable of addressing its multifactorial nature.

In this clinical practice, patient engagement and follow-up emerged as central considerations. Approximately half of patients did not return for a second visit, reflecting the advanced disease burden, competing clinical priorities, and care transitions common in this population. However, there was no difference in the population of patients that returned for at least one follow-up visit versus those that did not return for follow up with respect to baseline patient demographics, functional parameters, baseline lab values as well as rates of weight loss across the three intervals. The attrition rate here is consistent with attrition rates in supportive/palliative oncology trials, which experience an attrition rate of 44% according to one review [38]. Thus, rather than representing a limitation unique to this clinic model, these patterns likely reflect the inherent challenges of delivering longitudinal cachexia care in a medically complex population. Importantly, they provide insight into how multidisciplinary cachexia services function in real-world practice outside controlled trial settings. Future work is needed to better define strategies that enhance sustained engagement and support patients throughout the trajectory of advanced disease.

Among patients who returned for follow-up, we observed stabilization of median weight and a statistically significant deceleration in weight loss trajectory following clinic entry, in line with prior studies [17,18,19,20,21]. This shift, from a median loss of −0.5 kg/month prior to enrollment to 0.0 kg/month after three months, occurred in a medically complex cohort with heterogeneous causes of weight loss. Given the retrospective design and lack of a comparator group, these findings should be interpreted as descriptive patterns rather than evidence of treatment effect.

These data help provide context against which emerging pharmacologic therapies may be interpreted. For example, ponsegromab, a monoclonal antibody targeting GDF-15, increased weight by 0.7 kg/month over 3 months in phase II trials with associated functional improvements [39], representing an effect beyond what is typically observed with current outpatient care.

Previous work has shown that hand grip strength and sit-to-stand times are prognostic indicators in patients with cachexia [40,41]. We found that the stabilization of body weight in our population was accompanied by a significant improvement in physical performance (SST times), but not strength (hand grip). The observed improvement in sit-to-stand performance, despite unchanged grip strength, may reflect enhanced neuromuscular coordination or endurance rather than isolated strength gains. Importantly, functional improvements do not necessarily reflect gains in muscle mass or true strength, particularly in the absence of direct body composition measurements. Recent data from a physiatry-led outpatient cancer cachexia rehabilitation program identified gluteal weakness as a common and clinically relevant impairment among patients with cachexia, highlighting the importance of proximal lower-extremity function in tasks such as repeated sit-to-stand performance [42]. Additionally, the handgrip test may not be sensitive enough to capture changes in this time frame or serve as a good proxy for functional independence, as others have mentioned [43,44]. Nevertheless, maintenance of hand grip strength, as opposed to loss, may in and of itself represent a favorable outcome in a condition where no standard of care exists.

Intervention-level associations should be interpreted cautiously, as these analyses were exploratory and confounded by indication. Among the specific interventions, the use of a home exercise prescription demonstrated the strongest association with weight gain (β = +1.988, *p* = 0.079), though this did not reach statistical significance. The evidence for exercise, either alone or in combination with other treatment modalities, remains mixed. Exercise therapy shows mechanistic evidence for mitigating cancer cachexia by preserving muscle mass, reducing inflammation, and improving metabolic function [45,46]. However, current human clinical evidence shows no consistent improvements in lean mass, strength, or quality of life [47].

In contrast, patients receiving protein powder supplementation with or without creatine experienced greater weight loss (β = −2.102) in multivariate testing. Increased protein intake, above 1.0–2.0 g/kg/day, is recommended to help mitigate muscle depletion, with some evidence suggesting that specific amino acids, such as leucine, may enhance muscle protein synthesis [48]. However, in clinical trials, there is no robust evidence that protein supplementation improves outcomes [16]. The impact of protein supplementation on subsequent energy intake and long-term appetite regulation requires further study in larger sample sizes.

In routine clinical practice, interventions were not randomly assigned but selected based on clinical judgment, symptom burden, disease trajectory, and patient preferences. As such, patients receiving protein supplementation or appetite stimulants may have represented those with more severe anorexia, greater inflammatory burden, or more advanced disease. Similarly, patients able to participate in home exercise prescriptions may have had better baseline functional reserve or lower disease severity. These factors introduce substantial confounding by indication that cannot be fully addressed in a retrospective observational model of this size. Accordingly, these regression findings should not be interpreted as evidence of benefit or harm but rather as exploratory observations that may inform future prospective study design.

This study has several important limitations. First, all longitudinal findings must be interpreted within the context of a retrospective, single-center observational design without a control group. Accordingly, temporal associations between clinic participation and weight or functional trajectories cannot establish causality. Observed improvements after clinic entry may reflect natural disease fluctuation, concurrent oncologic or medical therapies, or selective follow-up of patients with more stable disease. We did not formally assess underlying disease burden or treatment changes, both of which may influence weight dynamics. Referral patterns may have introduced selection bias, as patients referred to our clinic may have had higher baseline engagement with care or greater access to support services. Furthermore, longitudinal and paired analyses were restricted to patients who survived and returned for follow-up, introducing potential survivorship bias. Patients who were clinically stable or more engaged may have been more likely to return, whereas those with aggressive disease trajectories, rapid decline, or transition to hospice are likely underrepresented in follow-up analyses. Body composition was not measured, limiting interpretation of whether weight stabilization reflected preservation of lean mass, fat mass, or fluid shifts. Clinic visits, laboratory testing, and interventions were billed as standard endocrinology care without dedicated external funding, which may limit generalizability to uninsured or under-resourced settings. Formal patient-reported outcome instruments were not systematically collected; symptoms such as appetite, fatigue, and functional limitation were documented clinically but not quantified using validated tools. Finally, inclusion of patients without malignancy introduces cohort heterogeneity and may limit direct comparison with oncology-specific cachexia cohorts. However, this reflects real-world referral patterns and supports the broader applicability of this clinic model beyond cancer-specific settings.

Still, as novel therapies targeting cachexia enter clinical trials, understanding the impact of current best-practice care is essential for contextualizing efficacy. Our study offers a pragmatic model for care delivery and a foundation for evaluating future interventions in routine clinical practice. Even modest gains in function or stabilization of decline, when achieved consistently, may represent meaningful progress in a field long defined by therapeutic inertia.

## 5. Conclusions

This study demonstrates the feasibility and real-world implementation of an endocrinology-led, multidisciplinary outpatient clinic for the management of unintentional weight loss within routine academic practice. In a medically complex and heterogeneous cohort, clinic participation was associated with stabilization of weight trajectories and improvement in functional performance among patients who returned for follow-up. These findings highlight both the challenges and the clinical value of delivering structured, individualized cachexia care outside of controlled trial settings. While causal inferences cannot be drawn, our data provide an important benchmark for standard outpatient management and underscore the potential role of endocrinology in advancing integrated, mechanism-informed care models. As emerging pharmacologic therapies are developed, scalable multidisciplinary frameworks such as this may serve as essential platforms for translating insights into meaningful functional outcomes for patients with cachexia.

## Figures and Tables

**Table 1:** Patient Demographics.

Parameters	N	%
**Race**		
White	67	65.0
African American	10	9.7
Asian	3	2.9
Other	12	11.7
Declined	11	10.7
**Sex**		
Male	55	53.4
Female	48	46.6
**BMI (kg/m^2^)**		
Underweight [<18.5]	25	24.5
Normal Weight [18.5–24.9]	55	53.9
Overweight [25–29.9]	19	18.6
Obese [>30]	3	2.9
**PNI**		
Severe Malnutrition	25	30.9
Moderate Malnutrition	24	29.6
Mild Malnutrition	17	21.0
Normal	15	18.5
**Cancer**		
Yes	66	64.1
No	37	35.9
**Metastasis**		
Yes	36	61.0
No	23	39.0
**Cancer type**		
Lung	12	17.1
Pancreatic	9	12.9
Esophageal	8	11.4
Head and Neck	4	5.7
Prostate	4	5.7
Lymphoma	3	4.3
Leukemia	3	4.3
Colorectal	3	4.3
Upper GI	3	4.3
Neuroendocrine	3	4.3
Breast	3	4.3
Urothelial	2	2.9
Glioblastoma	2	2.9
Thyroid	2	2.9
Renal	2	2.9
Other	7	10.0
**Diabetes**		
Yes	24	23.3
No	79	76.7
**Thyroid Disease**		
Yes	30	29.1
No	73	70.9

**Table 2:** Diagnoses and Interventions.

	N	%
**Diagnosis (ICD code)**		
Abnormal weight loss (R63.4)	69	67.0
Cachexia (R64.0)	47	45.6
Anorexia (R63.0)	35	34.0
Sarcopenia (M62.84)	27	26.2
Moderate protein-calorie malnutrition (E44.0)	20	19.4
Unspecified protein-calorie malnutrition (E46.0)	12	11.7
Feeding difficulties (R63.3)	2	1.9
**Intervention (Yes)**		
Protein/Calorie increase	79	76.7
Protein Powder (w/ and w/o creatine)	68	66.0
Exercise Videos	62	60.2
Appetite Stimulant	30	29.1
Anabolic Steroids/Hormone Therapy	19	18.4
Pancreatic Enzyme Replacement	13	12.6

**Table 3:** Participant weights at different timepoints relative to intervention.

Timepoint	N	Median [IQR]
−12 months	66	62.9 [52.8–76.0]
−6 months	71	65.0 [53.3–79.3]
0 months	103	60.3 [49.7–74.3]
3 months	80	59.2 [48.5–71.9]

**Table 4:** Pairwise Comparison of Rate of Weight Change.

Comparison	N (Pairs)	Raw *p*-Value	Adjusted *p*-Value *
−12 to −6 months vs. −6 months to 0 months	47	0.171	0.513
−6 to 0 months vs. 0 to 3 months	47	1.25 × 10^−5^	0.000038
−12 to −6 months vs. 0 to 3 months	47	0.011	0.033

* Bonferroni correction for multiple comparisons.

## Data Availability

Some or all datasets generated during and/or analyzed during the current study are not publicly available but are available from the corresponding author on reasonable request.

## Supplementary Materials

The following supporting information can be downloaded at: https://www.mdpi.com/article/10.3390/cancers18060946/s1, Table S1. Participant weights at different time points relative to intervention for 47 patients with available paired data; Table S2. Patient Demographics amongst clinic returners and non-returners; Table S3. Rate of weight change by time point amongst clinic returners and non-returners; Table S4. Initial functional parameters amongst clinic returners and non-returners; Table S5. Multiple Linear Regression Results: Effects of Intervention on Weight Change; Figure S1: Baseline lab values amongst patients that returned to clinic for at least one clinic visit versus those that did not return to clinic; Figure S2: Associations between baseline inflammatory biomarkers and percentage weight change; Figure S3: Associations between baseline hematologic parameters and percentage weight change; Figure S4: Associations between baseline metabolic and hormonal markers and percentage weight change.

## References

[1] Ferrer M., Anthony T., Ayres J., Biffi G., Brown J., Caan B., Feliciano E., Coll A., Dunne R., Goncalves M. (2023). Cachexia: A systemic consequence of progressive, unresolved disease. Cell.

[2] von Haehling S., Anker S. (2014). Prevalence, incidence and clinical impact of cachexia: Facts and numbers—Update 2014. J. Cachexia Sarcopenia Muscle.

[3] Wheelwright S., Darlington A., Hopkinson J., Fitzsimmons D., Johnson C. (2015). A systematic review and thematic synthesis of quality of life in the informal carers of cancer patients with cachexia. Palliat. Med..

[4] Quinten C., Coens C., Mauer M., Comte S., Sprangers M., Cleeland C., Osoba D., Bjordal K., Bottomley A. (2009). Baseline quality of life as a prognostic indicator of survival: A meta-analysis of individual patient data from EORTC clinical trials. Lancet Oncol..

[5] Baracos V., Martin L., Korc M., Guttridge D., Fearon K. (2018). Cancer-associated cachexia. Nat. Rev. Dis. Primers.

[6] Aversa Z., Costelli P., Muscaritoli M. (2017). Cancer-induced muscle wasting: Latest findings in prevention and treatment. Ther. Adv. Med. Oncol..

[7] Zhang Q., Song M., Zhang X., Ding J., Ruan G., Zhang X., Liu T., Yang M., Ge Y., Tang M. (2021). Association of systemic inflammation with survival in patients with cancer cachexia: Results from a multicentre cohort study. J. Cachexia Sarcopenia Muscle.

[8] Ni J., Zhang L. (2020). Cancer Cachexia: Definition, Staging, and Emerging Treatments. Cancer Manag. Res..

[9] Hariyanto T., Kurniawan A. (2021). Appetite problem in cancer patients: Pathophysiology, diagnosis, and treatment. Cancer Treat. Res. Commun..

[10] Iglesia D., Avci B., Kiriukova M., Panic N., Bozhychko M., Sandru V., de-Madaria E., Capurso G. (2020). Pancreatic exocrine insufficiency and pancreatic enzyme replacement therapy in patients with advanced pancreatic cancer: A systematic review and meta-analysis. United Eur. Gastroenterol. J..

[11] Bae J., Park J., Yang H., Kim J. (1998). Nutritional status of gastric cancer patients after total gastrectomy. World J. Surg..

[12] Larsen H., Krogh K., Borre M., Gregersen T., Hansen M., Arveschoug A., Christensen P., Drewes A., Emmertsen K., Laurberg S. (2023). Chronic loose stools following right-sided hemicolectomy for colon cancer and the association with bile acid malabsorption and small intestinal bacterial overgrowth. Color. Dis..

[13] Gee C., Fleuret C., Wilson A., Levine D., Elhusseiny R., Muls A., Cunningham D., Kohoutova D. (2021). Bile Acid Malabsorption as a Consequence of Cancer Treatment: Prevalence and Management in the National Leading Centre. Cancers.

[14] Bahardoust M., Mousavi S., Ziafati H., Alipour H., Haghmoradi M., Olamaeian F., Tayebi A., Tizmaghz A. (2024). Vitamin B12 deficiency after total gastrectomy for gastric cancer, prevalence, and symptoms: A systematic review and meta-analysis. Eur. J. Cancer Prev..

[15] Wyart E., Bindels L., Mina E., Menga A., Stanga S., Porporato P. (2020). Cachexia, a Systemic Disease beyond Muscle Atrophy. Int. J. Mol. Sci..

[16] Roeland E., Bohlke K., Baracos V., Bruera E., Del Fabbro E., Dixon S., Fallon M., Herrstedt J., Lau H., Platek M. (2020). Management of Cancer Cachexia: ASCO Guideline. J. Clin. Oncol..

[17] Bland K., Harrison M., Zopf E., Sousa M., Currow D., Ely M., Agar M., Butcher B., Vaughan V., Dowd A. (2021). Quality of Life and Symptom Burden Improve in Patients Attending a Multidisciplinary Clinical Service for Cancer Cachexia: A Retrospective Observational Review. J. Pain Symptom Manag..

[18] Del Fabbro E., Hui D., Dalal S., Dev R., Nooruddin Z., Bruera E. (2011). Clinical outcomes and contributors to weight loss in a cancer cachexia clinic. J. Palliat. Med..

[19] Del Fabbro E., Orr T., Stella S. (2017). Practical approaches to managing cancer patients with weight loss. Curr. Opin. Support. Palliat. Care.

[20] Parmar M., Vanderbyl B., Kanbalian M., Windholz T., Tran A., Jagoe R. (2017). A multidisciplinary rehabilitation programme for cancer cachexia improves quality of life. BMJ Support. Palliat. Care.

[21] Vigano A., Del Fabbro E., Bruera E., Borod M. (2012). The cachexia clinic: From staging to managing nutritional and functional problems in advanced cancer patients. Crit. Rev. Oncog..

[22] Jones S., Kon S., Canavan J., Patel M., Clark A., Nolan C., Polkey M., Man W. (2013). The five-repetition sit-to-stand test as a functional outcome measure in COPD. Thorax.

[23] Clinical Resources|STEADI—Older Adult Fall Prevention. https://www.cdc.gov/steadi/hcp/clinical-resources/index.html.

[24] Patel S., Duchowny K., Kiel D., Correa-de-Araujo R., Fielding R., Travison T., Magaziner J., Manini T., Xue Q., Newman A. (2020). Sarcopenia Definition & Outcomes Consortium Defined Low Grip Strength in Two Cross-Sectional, Population-Based Cohorts. J. Am. Geriatr. Soc..

[25] Vilarinho R., Montes A., Noites A., Silva F., Melo C. (2024). Reference values for the 1-minute sit-to-stand and 5 times sit-to-stand tests to assess functional capacity: A cross-sectional study. Physiotherapy.

[26] Gao S., Xia Y., Wu Q., Chang Q., Zhao Y. (2021). Reference Values for Five-Repetition Chair Stand Test Among Middle-Aged and Elderly Community-Dwelling Chinese Adults. Front. Med..

[27] Bohannon R. (2006). Reference values for the five-repetition sit-to-stand test: A descriptive meta-analysis of data from elders. Percept. Mot. Ski..

[28] Bhasin S., Brito J., Cunningham G., Hayes F., Hodis H., Matsumoto A., Snyder P., Swerdloff R., Wu F., Yialamas M. (2018). Testosterone Therapy in Men with Hypogonadism: An Endocrine Society Clinical Practice Guideline. J. Clin. Endocrinol. Metab..

[29] LeFevre M. (2015). Screening for thyroid dysfunction: U.S. Preventive Services Task Force recommendation statement. Ann. Intern. Med..

[30] Ridker P. (2016). A Test in Context: High-Sensitivity C-Reactive Protein. J. Am. Coll. Cardiol..

[31] Myers G., Rifai N., Tracy R., Roberts W., Alexander R., Biasucci L., Catravas J., Cole T., Cooper G., Khan B. (2004). CDC/AHA Workshop on Markers of Inflammation and Cardiovascular Disease: Application to Clinical and Public Health Practice: Report from the laboratory science discussion group. Circulation.

[32] Said E., Al-Reesi I., Al-Shizawi N., Jaju S., Al-Balushi M., Koh C., Al-Jabri A., Jeyaseelan L. (2021). Defining IL-6 levels in healthy individuals: A meta-analysis. J. Med. Virol..

[33] Cava F., González C., Pascual M., Navajo J., González-Buitrago J. (2000). Biological variation of interleukin 6 (IL-6) and soluble interleukin 2 receptor (sIL2R) in serum of healthy individuals. Cytokine.

[34] Forget P., Khalifa C., Defour J., Latinne D., Van Pel M., De Kock M. (2017). What is the normal value of the neutrophil-to-lymphocyte ratio?. BMC Res. Notes.

[35] Song M., Graubard B., Rabkin C., Engels E. (2021). Neutrophil-to-lymphocyte ratio and mortality in the United States general population. Sci. Rep..

[36] Wang Q., Jiang Y., Jin F., Gan L., Li R., Sun D., Zhang M., Pei Z., Zhang J., Ye J. (2025). Reference range of neutrophil-to-lymphocyte ratio in healthy individuals and its predictive value for post-trauma nosocomial infections. Front. Cell. Infect. Microbiol..

[37] Akirov A., Masri-Iraqi H., Atamna A., Shimon I. (2017). Low Albumin Levels Are Associated with Mortality Risk in Hospitalized Patients. Am. J. Med..

[38] Hui D., Glitza I., Chisholm G., Yennu S., Bruera E. (2012). Attrition rates, reasons and predictive factors in supportive and palliative oncology clinical trials. Cancer.

[39] Groarke J., Crawford J., Collins S., Lubaczewski S., Roeland E., Naito T., Hendifar A., Fallon M., Takayama K., Asmis T. (2024). Ponsegromab for the Treatment of Cancer Cachexia. N. Engl. J. Med..

[40] Xie H., Ruan G., Wei L., Zhang H., Ge Y., Zhang Q., Lin S., Song M., Zhang X., Liu X. (2023). Hand grip strength-based cachexia index as a predictor of cancer cachexia and prognosis in patients with cancer. J. Cachexia Sarcopenia Muscle.

[41] Fukushima T., Katsushima U., Ogushi N., Hase K., Nakano J. (2025). Lower-extremity muscle strength is associated with prognosis in patients with advanced or recurrent lung cancer: A retrospective, observational study. BMC Cancer.

[42] Dong K., Abplanalp K., Roy I. (2025). Cancer Cachexia Rehabilitation in a Novel Outpatient Clinical Program. Am. J. Phys. Med. Rehabil..

[43] Sawaya Y., Ishizaka M., Hirose T., Shiba T., Onoda K., Kubo A., Maruyama H., Urano T. (2021). Minimal detectable change in handgrip strength and usual and maximum gait speed scores in community-dwelling Japanese older adults requiring long-term care/support. Geriatr. Nurs..

[44] Barber A., Willbanks A., Abplanalp K., Lewis C., Binder-Markey B., Jayabalan P., Lieber R., Roy I. (2025). Six-Minute Walk Test Is Superior to Grip Strength as a Marker of Functional Recovery During Cancer Cachexia Rehabilitation. J. Cachexia Sarcopenia Muscle.

[45] Tsitkanou S., Murach K., Washington T., Greene N. (2022). Exercise Counteracts the Deleterious Effects of Cancer Cachexia. Cancers.

[46] Morinaga M., Sako N., Isobe M., Lee-Hotta S., Sugiura H., Kametaka S. (2021). Aerobic Exercise Ameliorates Cancer Cachexia-Induced Muscle Wasting Through Adiponectin Signaling. Int. J. Mol. Sci..

[47] Grande A., Silva V., Neto L.S., Basmage J., Peccin M., Maddocks M. (2021). Exercise for cancer cachexia in adults. Cochrane Database Syst. Rev..

[48] Antoun S., Raynard B. (2018). Muscle protein anabolism in advanced cancer patients: Response to protein and amino acids support, and to physical activity. Ann. Oncol..

