# Supplementary material for: Implementation and Feasibility of a Multidisciplinary Endocrine-Led Outpatient Clinic for Cancer Cachexia and Other Forms of Unintentional Weight Loss: A Real-World Observational Study
Source: Cancers (Basel). 2026 Mar 13;18(6):946. doi: 10.3390/cancers18060946 (PMC13026067; doi:10.3390/cancers18060946)
Supplement: Supplementary file 1 [file cancers-18-00946-s001.zip › cancers-4149268-supplementary.pdf]

Supplementary Figures and Tables

Supplementary Table S1. Participant weights at different time points relative to intervention for 47 patients with available paired data

Timepoint	*Median [IQR]*
-12 months	*64.0 [53.4-79.7]*
-6 months	*63.9 [52.6-78.5]*
0 months	*58.5 [49.0-74.3]*
3 months	*59.2 [49.2-74.2]*

Supplementary Table S2. Patient Demographics amongst clinic returners and non-returners

	**Patients with 1 or more follow-ups**		**Patients with no follow-ups**		**Fisher's exact Test**
**Parameters**	**N**	**%**	**N**	**%**	***p*-value**
**Race**					
White	32	62.7	35	67.3	
African American	4	7.8	6	11.5	
Asian	1	2.0	2	3.8	0.429
Other	9	17.6	3	5.8	
Declined	5	9.8	6	11.5	
**Sex**					
Male	25	49	30	57.7	0.4322
Female	26	51	22	42.3	
**BMI (kg/m^2)**					
Underweight [<18.5]	11	21.6	14	27.5	
Normal Weight [18.5-24.9]	32	62.7	24	47.1	0.4859
Overweight [25-29.9]	7	13.7	11	21.6	
Obese [>30]	1	2	2	3.9	
**PNI**					
Severe Malnutrition	11	25	14	37.8	
Moderate Malnutrition	11	25	13	35.1	0.2055
Mild Malnutrition	11	25	6	16.2	
Normal	11	25	4	10.8	
**Cancer**					
Yes	34	66.7	32	61.5	0.6824
No	17	33.3	20	38.5	
**Metastasis**					
Yes	19	65.5	17	56.7	0.7816
No	10	34.5	13	43.3	
**Cancer type**					
Lung	7	20.0	5	14.3	
Pancreatic	4	11.4	5	14.3	0.8198
Esophageal	4	11.4	4	11.4	

Head and Neck	1	2.9	3	8.6	
Prostate	2	5.7	2	5.7	
Lymphoma	2	5.7	1	2.9	
Leukemia	0	0.0	3	8.6	
Colorectal	2	5.7	1	2.9	
Upper GI	1	2.9	2	5.7	
Neuroendocrine	2	5.7	1	2.9	
Breast	1	2.9	2	5.7	
Urothelial	2	5.7	0	0.0	
Glioblastoma	1	2.9	1	2.9	
Thyroid	1	2.9	1	2.9	
Renal	2	5.7	0	0.0	
Other	3	8.6	4	11.4	
**Diabetes**					
Yes	11	21.6	13	25	0.8164
No	40	78.4	39	75	
**Thyroid Disease**					
Yes	16	31.4	14	26.9	0.6685
No	35	68.6	38	73.1	

Supplementary Table S3. Rate of weight change by time point amongst clinic returners and non-returners

	**Patients with 1 or more follow-ups**		**Patients with no follow-ups**		**Wilcox test**
**Timepoint**	**N**	**Median [IQR] kg/month**	**N**	**Median [IQR] kg/month**	***p*-value**
-12 to -6 months	29	-0.300 [-0.683-0.0333]	25	-0.833 [-0.683-0.150]	0.549
-6 months to 0 months	37	-0.417 [-0.933-0]	34	-0.675 [-1.59—0.0958]	0.0997
0 to 3 months	47	0.233 [-0.100-0.880]	33	0 [-0.433-0.367]	0.129

Supplementary Table S4. Initial functional parameters amongst clinic returners and non-returners

	**Patients with 1 or more follow-ups**		**Patients with no follow-ups**		**Wilcox test**
**Functional parameter**	**N**	**Median [IQR]**	**N**	**Median IQR**	***p*-value**
Initial max HGS (kg)	23	30 [19-35]	13	20 [18-40]	0.9473
Initial 5x SST (sec)	19	16.5 [12.6-20.5]	10	13.4 [11.-15.3]	0.1790

Supplementary Table S5. Multiple Linear Regression Results: Effects of Intervention on Weight Change

**Variable**	**Coefficient (B)**	**Standard Error**	**t-value**	***p*-value**
**Intercept**	-0.325	0.886	-0.367	0.715
Exercise Videos	+1.988	1.114	1.784	0.079[†]
Protein Powder (w/ or w/o Creatine)	-2.120	1.323	-1.603	0.113
Protein/Calorie Increase	+1.060	1.275	0.831	0.409
Anabolic Steroids/Hormone Therapy	-0.123	1.018	-0.121	0.904
Appetite Stimulant	+0.593	0.877	0.676	0.501
Creon	-0.012	1.195	-0.010	0.992

[†] $p < 0.1$

Baseline lab values amongst clinic returners and non-returners

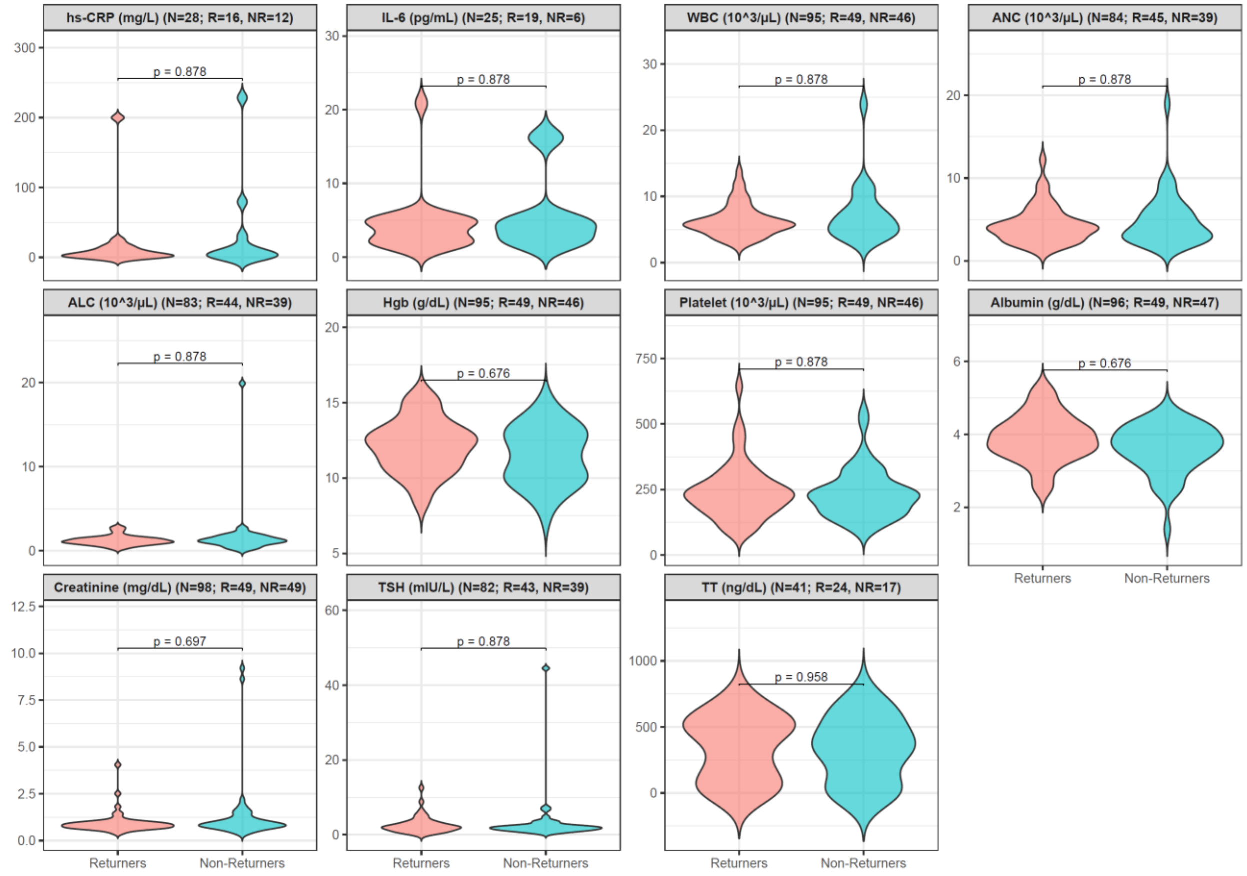

Supplementary Figure S1: Baseline lab values amongst patients that returned to clinic for at least one clinic visit versus those that did not return to clinic.

Violin plots depict the distribution of baseline laboratory values among patients who returned for at least one follow-up visit (Returners) and those who did not (Non-Returners). Parameters shown include high-sensitivity C-reactive protein (hs-CRP), interleukin-6 (IL-6), white blood cell count (WBC), absolute neutrophil count (ANC), absolute lymphocyte count (ALC), hemoglobin (Hgb), platelet count, albumin, creatinine, thyroid-stimulating hormone (TSH), and total testosterone (TT). Sample sizes for each comparison are indicated in the panel titles (total N, number of Returners [R], and Non-Returners [NR]). *p*-values displayed above each comparison were calculated using Wilcoxon rank-sum tests. No statistically significant differences were observed between groups for any baseline laboratory parameter.

Inflammatory Panel

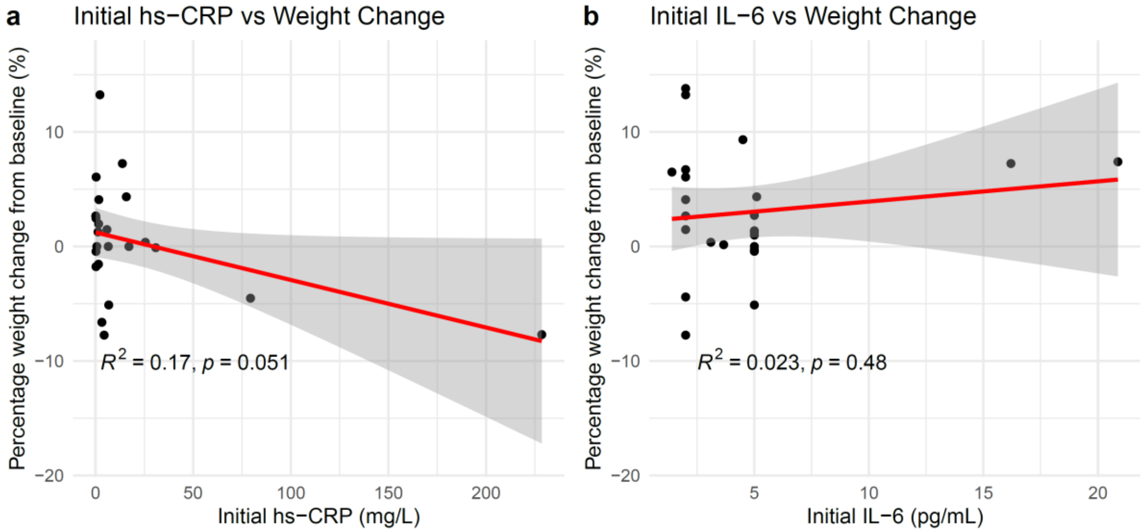

Supplementary Figure S2: Associations between baseline inflammatory biomarkers and percentage weight change.

Scatter plots show the relationships between (**a**) baseline hs-CRP (N=28) and (**b**) baseline IL-6 levels (N=25) with percentage weight change from baseline over the study period. Each point represents an individual participant. Red lines indicate linear regression fits, with shaded areas depicting 95% confidence intervals. Baseline hs-CRP showed a non-significant trend toward greater weight loss (R^2 = 0.17, p = 0.051), while no significant association was observed between baseline IL-6 and weight change (R^2 = 0.023, p = 0.48) using Spearman's rank correlation coefficient.

Hematology Panel

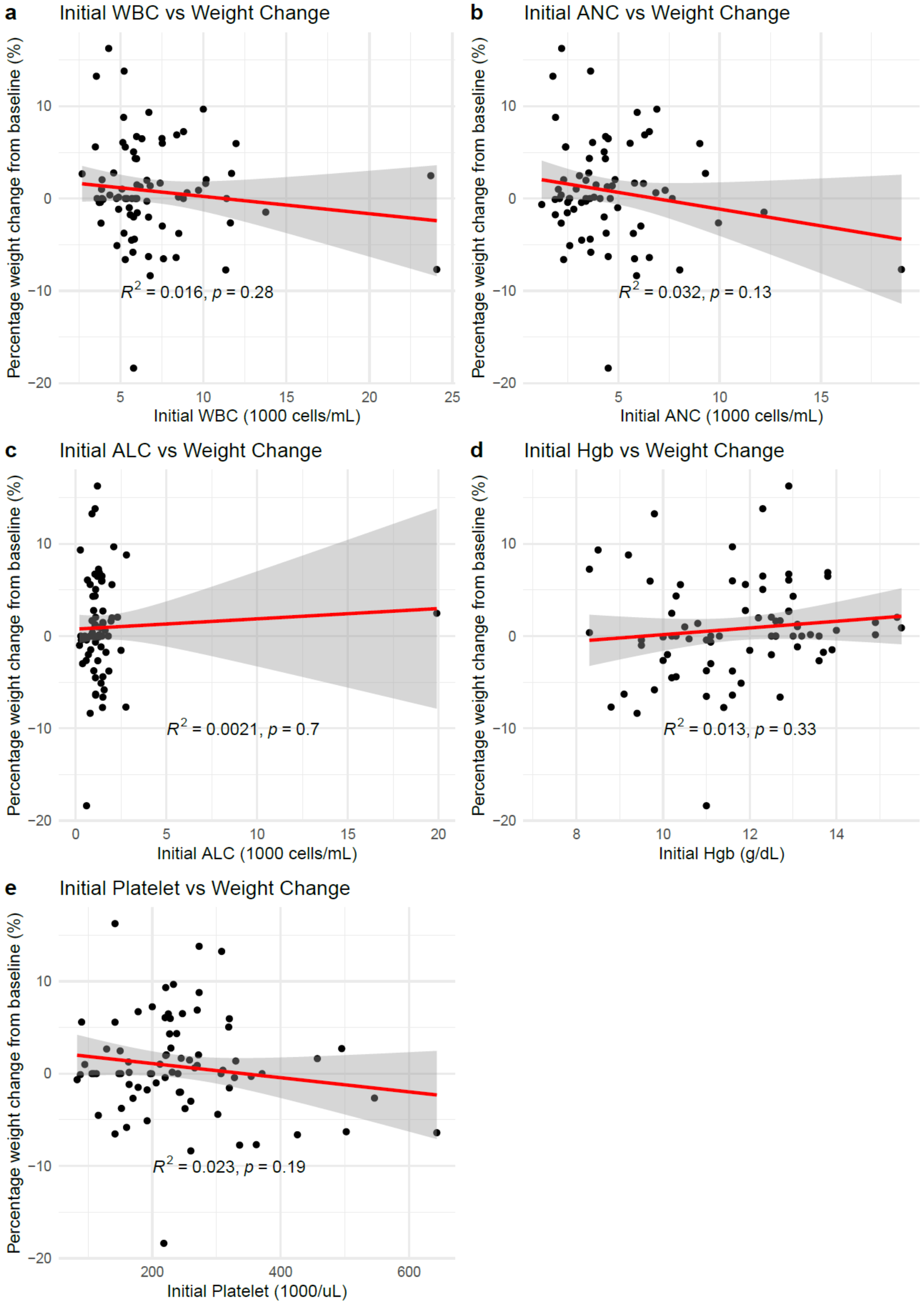

Supplementary Figure S3: Associations between baseline hematologic parameters and percentage weight change.

Scatter plots illustrate the relationships between baseline laboratory values and subsequent percentage change in body weight over the study period: (a) white blood cell count (WBC) (N=95), (b) absolute

neutrophil count (ANC) (N=84), (c) absolute lymphocyte count (ALC) (N=83), (d) hemoglobin (Hgb) (N=95), and (e) platelet count (N=95). Each point represents an individual participant. Red lines indicate linear regression fits, and shaded regions show 95% confidence intervals. No statistically significant associations were observed between any hematologic parameter and weight change using Spearman's rank correlation coefficient. R^2 and p-values for each regression are displayed within each panel.

Miscellanous Panel

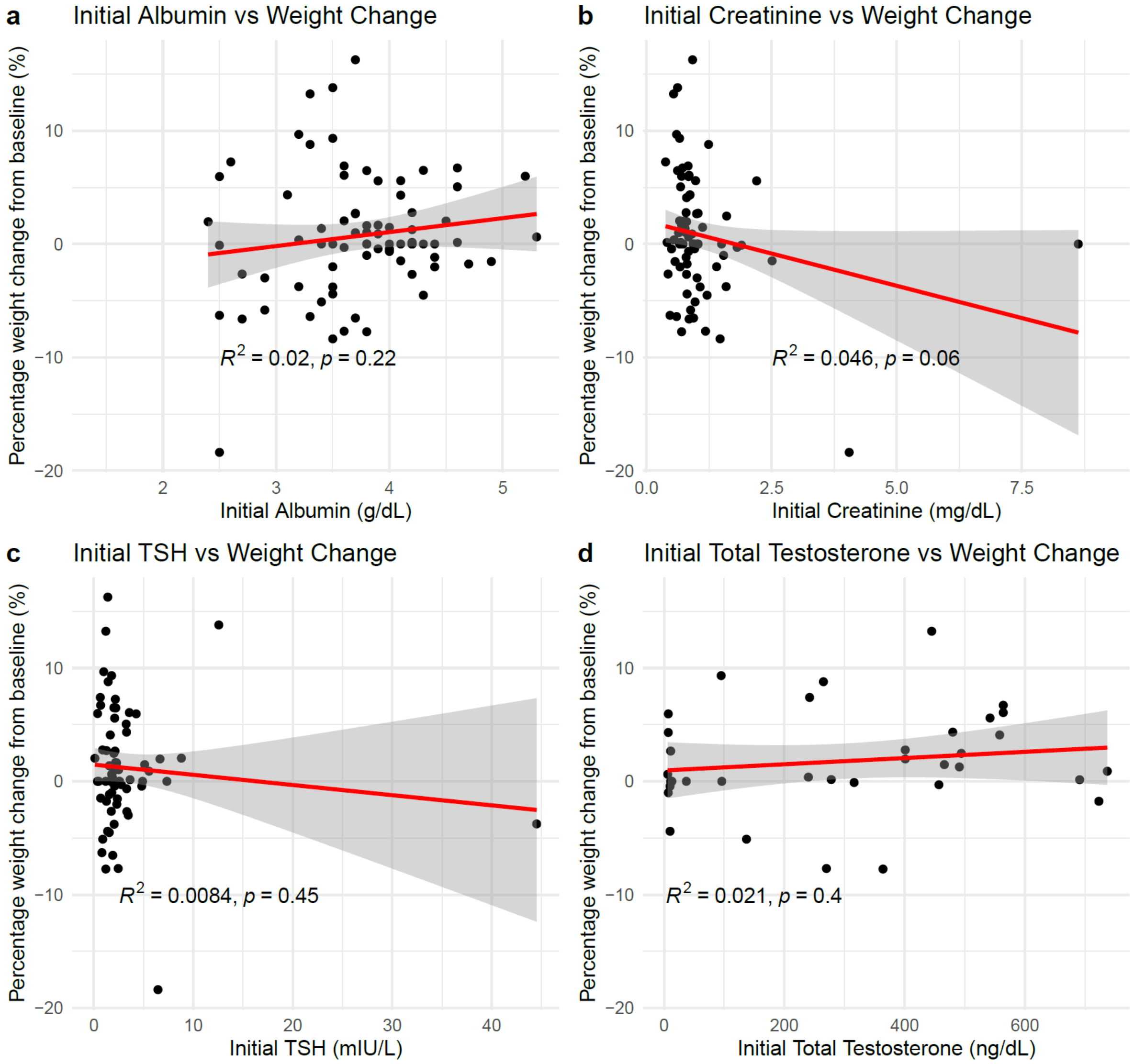

Supplementary Figure S4: ***Associations between baseline metabolic and hormonal markers and percentage weight change.***

Scatter plots depict the relationships between initial laboratory values and percentage change in body weight over the study period: (a) serum albumin (N=96), (b) serum creatinine (98), (c) thyroid-stimulating hormone (TSH) (N=82), and (d) total testosterone (N=41). Each point represents an individual participant. Red lines indicate linear regression fits, and shaded areas show 95% confidence intervals. No statistically significant associations were observed between these biomarkers and weight change using Spearman's rank correlation coefficient. R^2 and *p*-values for each regression are reported within each panel.
